# Microbial Communities Are Well Adapted to Disturbances in Energy Input

**DOI:** 10.1128/mSystems.00117-16

**Published:** 2016-09-13

**Authors:** Nuria Fernandez-Gonzalez, Julie A. Huber, Joseph J. Vallino

**Affiliations:** a The Josephine Bay Paul Center, Marine Biological Laboratory, Woods Hole, Massachusetts, USA; b Ecosystems Center, Marine Biological Laboratory, Woods Hole, Massachusetts, USA; Chinese Academy of Sciences

**Keywords:** 16S rRNA gene, bacteria, internal community dynamics, microbial community dynamics, chemostat cultures, endogenous drivers, energy input pulse, rare biosphere, structure and function

## Abstract

Although microbial systems are well suited for studying concepts in ecological theory, little is known about how microbial communities respond to long-term periodic perturbations beyond diel oscillations. Taking advantage of an ongoing microcosm experiment, we studied how methanotrophic microbial communities adapted to disturbances in energy input over a 20-day cycle period. Sequencing of bacterial 16S rRNA genes together with quantification of microbial abundance and ecosystem function were used to explore the long-term dynamics (510 days) of methanotrophic communities under continuous versus cyclic chemical energy supply. We observed that microbial communities appeared inherently well adapted to disturbances in energy input and that changes in community structure in both treatments were more dependent on internal dynamics than on external forcing. The results also showed that the rare biosphere was critical to seeding the internal community dynamics, perhaps due to cross-feeding or other strategies. We conclude that in our experimental system, internal feedbacks were more important than external drivers in shaping the community dynamics over time, suggesting that ecosystems can maintain their function despite inherently unstable community dynamics.

**IMPORTANCE** Within the broader ecological context, biological communities are often viewed as stable and as only experiencing succession or replacement when subject to external perturbations, such as changes in food availability or the introduction of exotic species. Our findings indicate that microbial communities can exhibit strong internal dynamics that may be more important in shaping community succession than external drivers. Dynamic “unstable” communities may be important for ecosystem functional stability, with rare organisms playing an important role in community restructuring. Understanding the mechanisms responsible for internal community dynamics will certainly be required for understanding and manipulating microbiomes in both host-associated and natural ecosystems.

## Abstract (precis)

Within the broader ecological context, biological communities are often viewed as stable and as only experiencing succession or replacement when subject to external perturbations, such as changes in food availability or the introduction of exotic species. Our findings indicate that microbial communities can exhibit strong internal dynamics that may be more important in shaping community succession than external drivers. Dynamic “unstable” communities may be important for ecosystem functional stability, with rare organisms playing an important role in community restructuring. Understanding the mechanisms responsible for internal community dynamics will certainly be required for understanding and manipulating microbiomes in both host-associated and natural ecosystems.

## INTRODUCTION

Microorganisms host a diverse repertoire of temporal strategies to maximize their productivity under a variety of environmental settings that undergo periodic as well as aperiodic change. Some strategies, such as circadian rhythms, require explicit molecular clocks for proper execution (1, 2), but clocks may also be present in nonphotosynthetic prokaryotes (3, 4). Bacteria can also exhibit anticipatory control (5), in which they respond to external cues, such as changes in temperature and oxygen concentration (6), to predict and adapt to environmental change before it occurs. Bacteria that anticipate environmental change have an obvious fitness advantage, and anticipatory strategies may stabilize ecosystems against perturbations (7, 8). Temporal strategies that do not rely on internal clocks include resource storage (9), hibernation and dormancy (10), and persister cells (11). Strategies organized over space can also increase fitness, such as diel vertical migration (12), luxury uptake (13), chemotaxis (14), and spatially executed redox reactions via cell gliding (15) or bacterial cables (16).

Temporal and spatial strategies have largely been studied in the context of an individual species’ or population’s fitness, even though such strategies can impart signatures to entire communities (17, 18), alter resource gradients that affect community function (19), and operate over a wide spectrum of scales (20). Our previous theoretical work based on nonequilibrium thermodynamics (21, 22) indicates that microbial systems capable of implementing temporal strategies (either actively or passively) can consume more food (i.e., energy) than communities that lack such strategies (23). Likewise, communities that can coordinate function over space can increase food acquisition relative to that of noncooperative communities (24). Since food acquired by a microorganism is ultimately respired by that organism or by a predator that eats it, an ecosystem that is near steady state can be characterized by the rate at which food (i.e., energy) is respired (i.e., energy dissipation). By accounting for microbial strategies that operate over time and space, the thermodynamic analysis provides a distinction between living and nonliving systems. Namely, nonliving systems maximize instantaneous energy dissipation, like a rock rolling down a hill, while the emergent behavior of living systems appears to maximize free energy dissipation when averaged over time and space due to spatial and temporal strategies that have been acquired by evolution. Temporal strategies allow living systems under certain conditions to outperform inanimate processes, but both appear to follow the same objective; they facilitate a systems race toward equilibrium (25).

To test our thermodynamic-based modeling approach and to explore how diverse microbial communities respond to temporally varying environments, we implemented a long-term microcosm experiment consisting of two control chemostats that received continuous input of energy in the form of methane and air and two treatment chemostats that received periodic energy inputs by cycling the feed gas between methane plus air and just air. The modeling work based on results from this experimental system was previously described in Vallino et al. (26), and the results indicated that temporal strategies over time scales equal to or longer than the cycle period resulted in greater energy. Furthermore, simulations using time scales shorter than the cycle period were unable to match experimental observations. However, this thermodynamic approach says little about the finer-scale community organization that gives rise to the larger-scale processes of energy dissipation or about the nature of the internal mechanisms that stabilize communities against external perturbations. These subjects are associated with long-standing questions in ecology on the nature of community structure versus ecosystem function and stability (27).

As recently reviewed by Song et al. (28), as well as by Shade et al. (29), there are numerous definitions associated with the concept of stability that are derived from the fields of physics and engineering that are used in ecology. However, even within a single ecosystem, it is possible to have subsystems that appear unstable, while higher-level components exhibit stability (30). Even the notion of stability itself is dependent on the time scale over which a system is observed (31). For example, an unstable system with a millennial time scale may appear stable if observed for only a year, but an unstable system with a monthly time scale will be perceived as unstable if observed for a year. The development of molecular tools has greatly improved observation of microbial systems, and several studies have now shown that microbial populations appear unstable, including populations in methanogenic communities (32), phytoplankton communities (33, 34), marine sediments (35), and nitrifying bioreactors (36, 37). Many of these systems exhibited functional stability even with unstable community dynamics, while in others, ecosystem function responded to community alterations. Functional complementarity (38, 39) can explain changes in the community composition for systems where external drivers were not or could not be held constant, but it is still uncertain what drives changes in community composition when external drivers are constant (40, 41).

To date, there has been limited research on the importance of internal dynamics relative to external drivers for changes in community composition, but the use of microbial microcosm experiments is well suited to address these questions, which can be challenging to address in field studies (42, 43). In this work, we used 16S rRNA gene sequencing together with quantification of microbial abundance and ecosystem function to explore the long-term dynamics (510 days) of a methanotrophic microbial community under both continuous and periodic energy inputs. The results suggest that microbial communities are inherently well adapted to disturbances in energy input, with the rare biosphere being critical to seeding internal community dynamics.

## RESULTS

### Ecosystem function.

**FIG 1:**
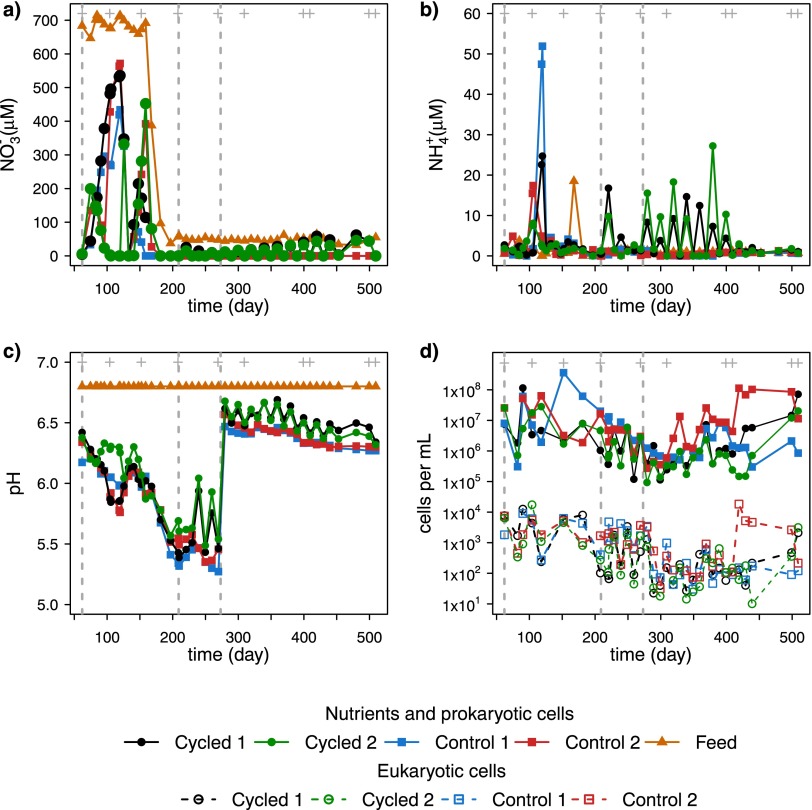
Environmental variables in chemostats and cell concentrations during phases II, III, and IV. (a) Nitrate and nitrite (NO_3_^−^), (b) ammonium (NH_4_^+^), (c) pH, and (d) prokaryotic and eukaryotic cell densities. Grey dashed lines indicate the start of phases II, III, and IV. Grey plus signs at the top of the panels indicate the days of microbial diversity measurements.

**FIG 2:**
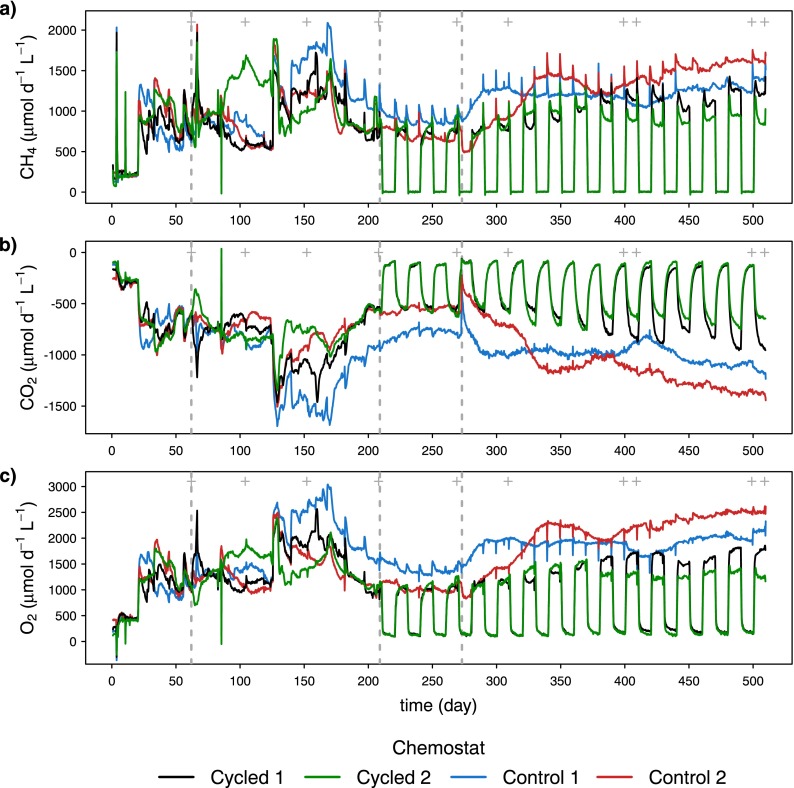
Gas production or consumption rates calculated from input and output gas concentrations and flow rate. (a) Methane consumption, (b) carbon dioxide production, and (c) oxygen consumption. Grey dashed lines indicate the start of phases II, III, and IV. Grey plus signs at the top of the panels indicate the days of microbial diversity measurements.

The experiment was divided into four phases. Initially, the methane-and-air mixture was kept constant during phases I and II. In phases III and IV, the chemostats were separated into control and cycled treatments, and while the control received a constant energy input, the cycled chemostats were subjected to cycling inputs of methane-and-air and air-only mixtures with a 20-day periodicity. To assess changes in ecosystem function across treatments, we characterized ecosystem processes by measuring the NH_4_^+^, NO_3_^−^, and NO_2_^−^ concentrations (hereinafter referred to as NO_3_), pH, prokaryote and eukaryote cell densities (Fig. 1), CH_4_ and O_2_ consumption and CO_2_ production rates (Fig. 2), total dissolved nitrogen (TDN), dissolved organic carbon (DOC) and nitrogen (DON), and particulate organic carbon (POC) and nitrogen (PON) (see Fig. S1 in the supplemental material). Gas consumption and production rates were calculated from differences in input and output gas concentrations (see Fig. S2) and flow rate.

10.1128/mSystems.00117-16.4 Figure S1  Environmental variables for microcosms and medium feed for phases II, III, and IV. (a) Dissolved organic carbon (DOC), (b) dissolved organic nitrogen (DON), and (c) total dissolved nitrogen (TDN). Grey dashed lines indicate the start of phases II, III, and IV. Download Figure S1, EPS file, 0.5 MB. Copyright © 2016 Fernandez-Gonzalez et al. 2016 Fernandez-Gonzalez et al. This content is distributed under the terms of the Creative Commons Attribution 4.0 International license.

10.1128/mSystems.00117-16.5 Figure S2  Gas concentrations. (a) Methane, (b) carbon dioxide, and (c) oxygen. Grey dashed lines indicate the start of phases II to IV. Download Figure S2, EPS file, 2 MB. Copyright © 2016 Fernandez-Gonzalez et al. 2016 Fernandez-Gonzalez et al. This content is distributed under the terms of the Creative Commons Attribution 4.0 International license.

Overall, most environmental variables in the cycled chemostats exhibited the influence of periodic energy inputs during experimental phases III and IV in the cycled chemostats, during which the measured values during CH_4_-on periods were similar to those in the control chemostats. For instance, nitrate and ammonia accumulated during the CH_4_-off periods, but when CH_4_ was on, their values were drawn down almost to 0 µM, close to the control measurements (Fig. 1a and b). In the last 100 days of the experiment, periodic accumulation of ammonium was not observed even though no changes in external drivers were made in phase IV (Fig. 1b).

The decreases in chemostat pH over phases I, II, and III (Fig. 1c) were likely caused by the increase in carbon dioxide concentration (see Fig. S2 in the supplemental material) and decrease in nitrate in the feed medium (Fig. 1a). In order to minimize losses to microbial community diversity, a 10 mM, pH 6.5 phosphate buffer was added to the feed medium on day 273, which defines the start of phase IV of the experiment. All other variables, except for the CH_4_ feed in cycled chemostats, were maintained constant during phase IV.

The eukaryotic and prokaryotic relative cell abundances exhibited nearly parallel behavior over the course of the entire experiment. Both cell densities fell when the pH decreased in phase III but later recovered during the first 150 days of phase IV (Fig. 1d). Biomass was present in the liquid and in biofilms inside all chemostats. To account for variation in biomass that could be influenced by biofilm production or sedimentation, samples were only taken after thorough homogenization of the chemostats via mixing (see Materials and Methods). Comparing the values across treatments, no significant loss of biomass was observed in the cycled chemostats even though they only received half of the energy input that the controls did in phases III and IV, where microbial cell abundances were similar within and between treatments.

Gas consumption and production rates in the control chemostats showed some long-term minor fluctuations and a tendency to increase or decrease slowly in phases III and IV but otherwise showed rather stable metabolic function (Fig. 2). In phases III and IV, the gas consumption and production rates in cycled chemostats were similar, although slightly lower or higher, to those observed in the controls during CH_4_-on periods. In addition, the recovery of CO_2_ gas production rates at the beginning of CH_4_-on periods was lagged compared to the CH_4_ and O_2_ rates due to carbonate chemistry dynamics, which was not accounted for in the rate calculations. The changes in gas rates observed during phases I and II were largely due to changes in operating conditions to prepare the systems for gas-cycling phases.

### Effects of energy input cycling on community richness and composition.

A total of 511,629 pyrosequencing sequences (7,984 to 17,288 per sample) spanning the V4-V6 region of the 16S rRNA bacterial gene were clustered into 18,610 operational taxonomic units (OTUs) at a 0.96 similarity cutoff (2,455 to 169 per sample). Overall, the library coverage indicated that three-quarters of the diversity was captured (see Table S1a in the supplemental material). We did not observe any treatment effect on bacterial richness or evenness estimations, although the values varied through time (see Table S1a). In particular, richness decreased and community unevenness increased in all chemostats when pH levels dropped to acidic values from late phase II until the beginning of phase IV (see Table S1a).

10.1128/mSystems.00117-16.2 Table S1  (a) Number of sequences, library coverage, and alpha diversity index for each sample. (b) PERMANOVA pairwise test results. Download Table S1, DOCX file, 0.1 MB. Copyright © 2016 Fernandez-Gonzalez et al. 2016 Fernandez-Gonzalez et al. This content is distributed under the terms of the Creative Commons Attribution 4.0 International license.

**FIG 3:**
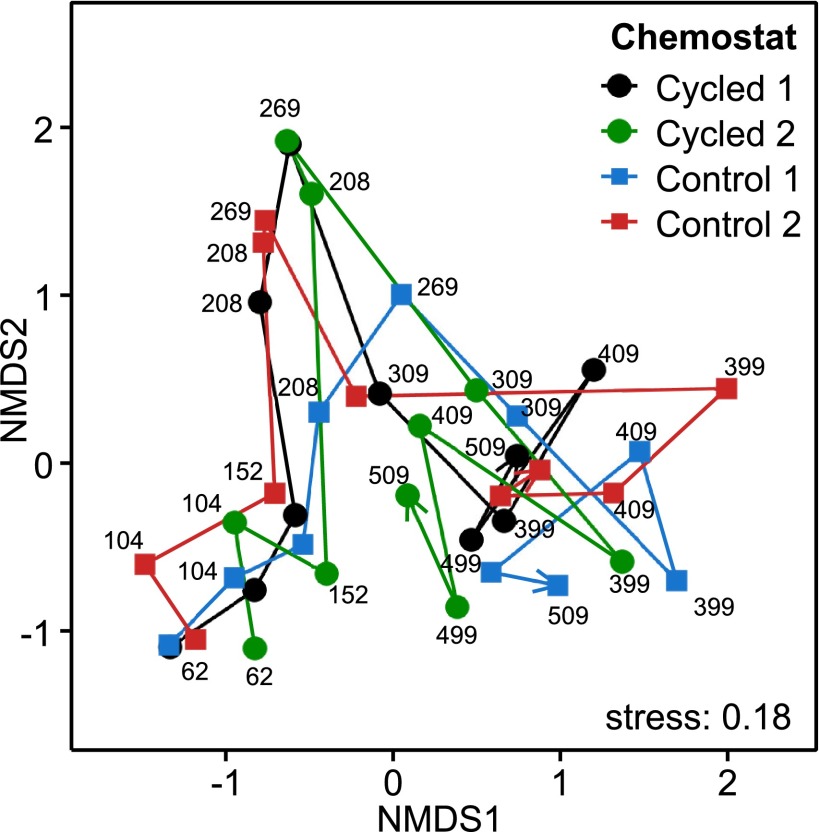
Community dissimilarities. Nonmetric multidimensional scaling (NMDS) ordination of the Morisita-Horn dissimilarity matrix among all bacterial communities. Samples from the same chemostat are connected with arrows indicating the timeline. Labels correspond to sampling times (days).

The Morisita-Horn (MH) dissimilarity index showed that communities shifted their composition throughout the experiment with no indication of greater community similarity within treatments than between treatments (Fig. 3). A permutational multivariate analysis of variance (PERMANOVA) test was used to examine whether bacterial community compositions within and between treatments were statistically different while accounting for the temporal trend. During phase II when the chemostats were being mixed, the communities changed in composition similarly, regardless of treatment (Fig. 3; see also Table S1b in the supplemental material). During both cycling phases, phases III and IV, the bacterial community composition was more dynamic and communities changed less similarly over time (overall test, *F* = 1.635, *P* = 0.196) (Fig. 3; see also Table S1b). The dynamic turnover of the microbial community was quite apparent when community dissimilarity between successive time points was examined in each chemostat separately (see Fig. S3). Except for the start (days 62 to 104) and during the low-pH phase III (days 208 to 269), communities differed considerably between two successive time points regardless of treatment.

10.1128/mSystems.00117-16.6 Figure S3  Community turnover. Morisita-Horn dissimilarities between communities from consecutive days within each microcosm. Grey dotted lines indicate the start of phases II to IV. Download Figure S3, EPS file, 0.2 MB. Copyright © 2016 Fernandez-Gonzalez et al. 2016 Fernandez-Gonzalez et al. This content is distributed under the terms of the Creative Commons Attribution 4.0 International license.

The communities were dominated by the phylum *Proteobacteria*, which averaged 70.75 and 71.47% of the community in cycled and control chemostats, respectively (see Fig. S4 in the supplemental material). The most abundant class within the phylum was *Gammaproteobacteria* (44.90% in cycled chemostats and 45.94% in control chemostats), although classes *Alphaproteobacteria* (9.76 and 11.80%) and *Betaproteobacteria* (13.90 and 12.10%) and phyla *Bacteroidetes* (8.14 and 8.28%) and *Verrucomicrobia* (4.52 and 3.61%) represented substantial percentages of the communities as well.

10.1128/mSystems.00117-16.7 Figure S4  Community composition at high taxonomic levels. Relative abundances of phyla for each microcosm through time*. Proteobacteria* phylum has been divided into its classes. Phyla *Proteobacteria*, *Bacteroidetes*, *Verrucomicrobia*, *Acidobacteria*, *Chloroflexi*, *Planctomycetes*, *Chlorobi*, *Cyanobacteria*, *Actinobacteria*, OP10, *Nitrospira*, *Gemmatimonadetes*, TM7, *Firmicutes*, BRC1, *Spirochaetes*, *Lentisphaerae*, *Fibrobacteres*, and OD1 are commonly found in freshwater environments. Download Figure S4, EPS file, 1.2 MB. Copyright © 2016 Fernandez-Gonzalez et al. 2016 Fernandez-Gonzalez et al. This content is distributed under the terms of the Creative Commons Attribution 4.0 International license.

**FIG 4:**
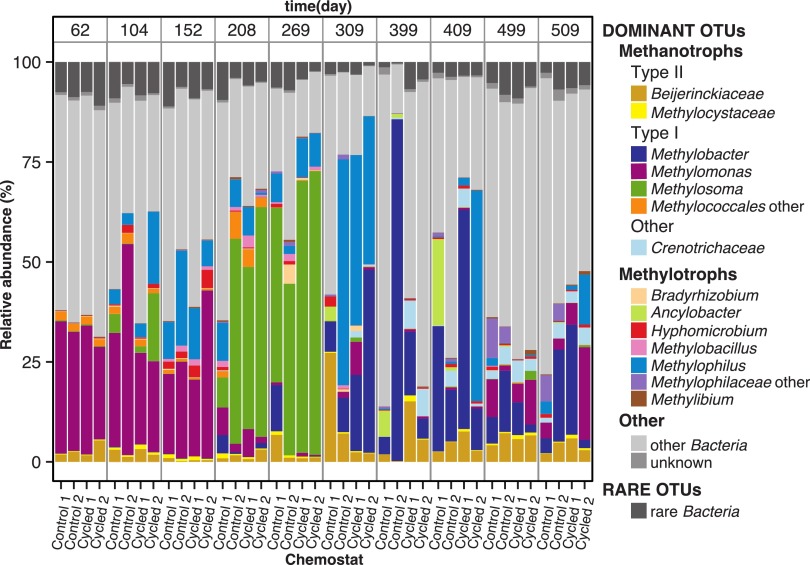
Cumulative relative abundances of dominant OTUs and rare OTUs (dark grey). For dominant OTUs, the relative abundances of one-carbon-degrading genera are indicated with colors other than grey. The “Other” category includes the rest of the dominant OTUs; specifically, either the remaining 66 *Bacteria* genera (see Table S2 in the supplemental material) or OTUs with unknown taxonomic classification. See the text for definitions of dominant and rare OTUs.

The OTUs were divided into two groups: dominant OTUs, defined as those with abundances equal to or over 1% in any of the samples analyzed in any chemostat, and rare OTUs, whose abundances were always below 1%. The 150 dominant OTUs represented over 90% of the community at almost all times across all chemostats (Fig. 4; see also Table S2 in the supplemental material). Most of the dominant OTUs (83%) were part of the community in all 4 chemostats (see Fig. S5). Linear discriminant analysis (LDA) effect size (LEfSe) was carried out between treatments for the cycling phases, phases III and IV, and found only 18 dominant OTUs (12%), which were distributed differentially across control and cycled treatments (see Fig. S6). In addition, microbial co-occurrence assemblage patterns were examined using network inference to infer associations between dominant OTUs and environmental variables. No significant correlations (Spearman’s > 0.6; *P* > 0.05) were found between OTUs and environmental variables. Only two large (over 5 nodes) clusters of co-occurring dominant OTUs were found, corresponding to OTUs that were abundant during phase II and phase IV, respectively (see Fig. S7).

10.1128/mSystems.00117-16.3 Table S2  Relative abundances of lower taxonomic groups that contain abundant OTUs. Download Table S2, PDF file, 0.1 MB. Copyright © 2016 Fernandez-Gonzalez et al. 2016 Fernandez-Gonzalez et al. This content is distributed under the terms of the Creative Commons Attribution 4.0 International license.

10.1128/mSystems.00117-16.8 Figure S5  Venn diagram of dominant OTUs shared between all microcosms during all experimental phases. Download Figure S5, EPS file, 0.2 MB. Copyright © 2016 Fernandez-Gonzalez et al. 2016 Fernandez-Gonzalez et al. This content is distributed under the terms of the Creative Commons Attribution 4.0 International license.

10.1128/mSystems.00117-16.9 Figure S6  Relative abundances of statistically significant differentiating OTUs for each treatment during phases III and IV based on LEfSe results. OTU label color indicates the treatment for which it is significant, as follows: cycled, green; control, blue. Data are averaged by day. Download Figure S6, EPS file, 0.6 MB. Copyright © 2016 Fernandez-Gonzalez et al. 2016 Fernandez-Gonzalez et al. This content is distributed under the terms of the Creative Commons Attribution 4.0 International license.

10.1128/mSystems.00117-16.10 Figure S7  Co-occurrence of dominant OTUs. (a) Network of dominant OTUs and environmental data. Only two highly interconnected groups of at least 5 co-occurring OTUs were found, cluster 1 and cluster 2. (b and c) Temporal variation of relative abundances for OTUs within cluster 1 (b) and cluster 2 (c) in each chemostat. Keys indicate the best taxonomy assignment and the identification number of each OTU. Download Figure S7, PDF file, 2.1 MB. Copyright © 2016 Fernandez-Gonzalez et al. 2016 Fernandez-Gonzalez et al. This content is distributed under the terms of the Creative Commons Attribution 4.0 International license.

### One-carbon-degrading bacteria and the rare biosphere.

One-carbon (C_1_)-degrading bacteria, both methanotrophs and methanol-degrading bacteria (hereinafter referred to as methylotrophs), were a large proportion of the community at most times (Fig. 4). These metabolic types were distributed across diverse genera, mostly within the type I methanotrophs of class *Gammaproteobacteria* (i.e., *Methylosoma* and *Methylobacter*). In addition, *Crenothrix* and putative type II methanotrophs from class *Alphaproteobacteria* (*Methylocystaceae* and *Beijerinkckiaceae* groups) were also present. Other C_1_ bacteria included putative methylotrophs from different families within class *Betaproteobacteria*, including *Methylophilaceae* (*Methylophilus* and *Methylobacillus*), but also *Bradyrhizobiaceae* (*Bradyrhizobium*), *Hyphomicrobiaceae* (*Hyphomicrobium* and *Ancylobacter*), and *Comamonadaceae* (*Methylibium*) (Fig. 4).

**FIG 5:**
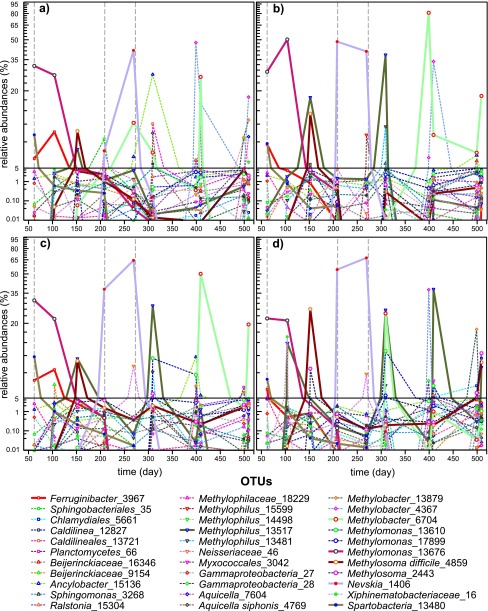
Temporal dynamics of highly dominant OTUs. Semilogarithmic plot of temporal changes in relative abundances of dominant OTUs whose relative abundances were larger than 5% in any of the samples in control (a, control 1; b, control 2) and cycled (c, cycled 1; d, cycled 2) microcosms. Solid lines identify the seven OTUs that were found at levels of 5% or greater in all four chemostats. Names correspond to the taxonomy and number of each OTU. Note the break in the *y* axis scales to highlight the importance of the rare biosphere. Vertical grey dashed lines indicate the start of phases II, III, and IV.

A succession of different OTUs belonging to genera in both methanotrophic and methylotrophic groups was observed over the course of the experiment (Fig. 4 and 5). The C_1_-degrading-genus successions were very similar across all chemostats during experiment phases II and III, but some divergence was observed during pH-controlled phase IV. Within the methanotrophs, we observed an initial dominance of *Methylomonas* (mainly OTUs 13610 and 17899) during I and II, which was replaced by *Methylosoma* (OTU 4859) when the pH was low at the end of phase II and phase III (Fig. 4 and 5). During phase IV, the chemostats were characterized by a more diverse and even distribution of methanotrophs, although overall, *Methylobacter* was the most dominant genus. This phase is also characterized by a small increase in type II methanotrophs and the appearance of *Crenothrix* (Fig. 4). The methylotrophs also changed with time. They were initially stimulated during early phase II, particularly *Methylophilus* (mainly OTU 15599), which was the most abundant methylotrophic genus, and then replaced by *Methylobacillus* under acidic conditions (Fig. 4 and 5). Later, in phase IV, the emergence of *Ancylobacter* and unclassified *Methylophilaceae* OTUs reconfigured the assembly of methylotrophs.

In all chemostats, each one of the abundant OTUs was also a member of the rare biosphere at certain times during the course of the experiment (Fig. 5). In particular, the changes in relative abundances were very large for the group of OTUs that represented more than 5% of the community in any of the samples analyzed (Fig. 5). For instance, *Methylosoma difficile*_4859 was a member of the rare biosphere on day 62 (<0.01%) in all chemostats, and at day 269, it represented over 40% of gene sequences (64, 67, 41, and 41% in chemostats cycled 1, cycled 2, control 1, and control 2, respectively). By day 399, it was back to <0.01% in all cases (Fig. 5). Other methanotrophs that were rare bacteria at the beginning of phase II, like *Methylobacter*_13879 (<0.01% in cycled and 8 × 10^−3^% in control chemostats), were at relatively high abundances at different times (50% at day 409 in cycled 1, 24% at day 309 in cycled 2, 26% at day 409 in control 1, and 81% at day 399 in control 2). Later, this OTU behaved differently depending on the chemostat: in some cases, *Methylobacter*_13879 stayed an abundant OTU for the rest of the experiment but exhibited large changes in relative abundance over short periods of time (9, 7, and 18% at days 409, 499, and 509, respectively, in control 2 and 4, 50, 2, and 20% at days 399, 409, 499, and 509, respectively, in cycled 1). In other cases, it dropped to the rare biosphere for almost or all the rest of the experiment (8 × 10^−3^%, 1%, 3 × 10^−2^%, and 0.4% at days 399, 409, 499, and 509 in cycled 2 and 0% at days 499 and 509 in control 1) (Fig. 5).

These types of changes were also observed for other highly abundant members of the community that are not C_1_ bacteria. In particular, *Aquicella siphonis*_4769 was a rare bacterium (0% in all chemostats from day 60 to 309) until day 399, when it represented 47, 6, and 37% of the community in control 1, control 2, and cycled 2, respectively. Later, *A. siphonis*_4769 progressively dropped to the rare biosphere again in cycled 2 (3, 1, and 0.01% at days 409, 499, and 509, respectively) but bounced back and forth in both control chemostats (18 to 4% in control 1 and 33 to 0.3% in control 2). In contrast, *A. siphonis*_4769 remained rare (0.1 to 9 × 10^−3^%) at all times in cycled 1.

## DISCUSSION

We used natural methanotrophic microbial microcosms to study how microbial communities respond to periodic inputs of energy by cycling inputs of methane and air mixtures. Overall, both the control and cycled chemostats were functionally stable. Both the nitrate and ammonium concentrations increased relative to the concentrations in the controls when methane was turned off, but the bacterial and protist cell counts were relatively stable during the cycling phases, phases III and IV, and there was no significant difference in cell counts between the control and cycled chemostats. Similarly, the methane oxidation rates in the control chemostats during phases III and IV were relatively constant, and while the methane oxidation rates in the cycled chemostats varied as a function of the gas inputs, there were no significant changes during phase IV. However, the bacterial community composition changed in both the control and cycled chemostats during all phases of the experiment. The community dynamics in the control chemostats were particularly striking during phase IV, given that the external drivers were maintained constant during that phase, and yet, the communities continued to show replacement of the dominant methanotroph at almost every time point. The cycled chemostats showed replacement of the dominant methanotroph at almost every sample point as well. In both control and cycled chemostats, the dominant OTU often originated from the rare biosphere (44) or was even undetected in the preceding sample.

Perhaps the most interesting result from this long-term perturbation experiment was the similarity in the community dynamics between the control and cycled chemostats. We expected that the cycled chemostats would develop a dramatically different microbial community that would be better adapted to cyclic energy inputs, but the results did not support this. Instead, we observed similarity in community composition succession between the control and cycled chemostats. Considering only the most abundant OTU at each sample point, seven of them were detected across all four chemostats, and they were the most abundant OTUs in 29 of the 40 samples examined. The speed at which the most abundant OTUs were replaced was most evident in the samples taken over a cycle period at days 399 (methane on), 409 (methane off), 499 (methane on), and 509 (methane off). It may not be surprising that the most abundant OTU was replaced between methane on (399 and 499) and methane off (409 and 509) in the cycled chemostats, but the switching also occurred in the controls even though the methane input was constant. Furthermore, the succession in dominance was opposite in the two control chemostats at day 399 and day 409, where dominance changed from OTU-4769 to OTU-13879 in control 1 and vice versa in control 2. Both of these OTUs dominated in the cycled chemostats as well, with OTU-13879 dominating at the end of methane off (day 409) and OTU-4769 dominating at the end of methane on (day 399). From these results, it appears that the internal feedbacks driving community dynamics were more important for shaping the community composition than the external drivers. Even though the cycled chemostats were significantly perturbed by periodic methane input, this external forcing was of minor importance for community dynamics and composition when referenced to the control chemostats.

Our observations are similar to those of others. Konopka et al. (45) studied 16 replicate microcosms subject to discrete pulses of gelatin every day and every 7 days and observed very dynamic bacterial communities, although they observed greater variability between their replicates than we did. Similar perturbation studies (46, 47) concluded that internal dynamics seemed to dominate and external forcing was not a strong selective pressure, which is consistent with our findings. Analysis of natural marine communities during a phytoplankton bloom also displayed rapid replacement of the dominant organisms and the importance of internal feedbacks in shaping communities (34). In pond microcosms, nutrient pulsing even stabilized ecosystem properties relative to those of nonpulsed controls via compensatory dynamics (48).

The lack of importance of external drivers in community dynamics implies that the microbial communities were inherently well adapted to periodic inputs of energy. If the microbial communities were not well adapted to interruptions in energy availability, then we would expect that the methane oxidation rates in the cycled chemostats would increase over time as the community adapted and evolved to the periodic availability of methane. This selection pressure, which was not present in the controls, would be expected to select for those organisms with enhanced resource storage capabilities that would allow growth and maintenance when methane was absent (9). Differential selection between chemostat treatments would drive changes in community composition and increases in methane oxidation rate over time. But neither of these outcomes was observed, which leads us to conclude that the communities were already well adapted to interruptions in energy input; there was little differential selection between chemostats because effective temporal strategies were already present in both treatments. Our previous modeling work (26) also supports the conclusion that the communities were well adapted to periodic inputs of energy, because the thermodynamically based optimal allocation model was only able to accurately simulate the observed methane oxidation rates in the control and cycled chemostats when the optimization interval (i.e., time scale of the implied temporal strategies) was set to be equal to or greater than the 20-day methane cycle period. When shorter optimization intervals were used, the model was unable to fit the observations (see Table 18.2 in reference 26). The observations from near the end of the experiment (day 1,242; data not shown) indicate that the temporal strategies were not clock based, because oscillations in gas dynamics were not observed in the cycled chemostats when the methane cycling was stopped. Such residual oscillations are observed in clock-based circadian systems when external cycling is terminated (49). The lack of residual oscillations when methane was left on indicates that the communities probably implemented passive temporal strategies, such as resource storage, which have been identified in methanotrophs that are known to store polyhydroxybutyrate under cyclic inputs (50), as well as fatty acids (51). If the external drivers were not responsible for the observed community dynamics in both the control and cycled chemostats, what might explain the internal dynamics?

Changes in community composition are often associated with changes in external drivers, as explained by functional complementarity (38) or compensatory dynamics in response to press or pulse perturbations (39). These theories have been put forth to explain the maintenance of biodiversity and why competitive exclusion (52) does not lead to the “paradox of the plankton” in which limited resources support a wide range of diversity (53). In complementarity, each species has evolved to grow maximally under a narrow set of environmental conditions, such as pH, temperature, light level, etc. As external drivers change the environment, such as by lowering the pH, succession in community composition follows, where those organisms that optimally match the new conditions are selected for, provided sufficient biodiversity exists within the system or can be readily imported by transport processes. The rare biosphere can serve as the reservoir for organisms whose traits are currently suboptimal under the prevailing conditions (41). Functional complementarity and similar theories may indeed explain the community succession we observed during phases I to III in both control and cycled chemostats due to changes in pH and in nutrient concentrations, where the ecosystem function of methane oxidation was maintained relatively stable by a succession of optimally adapted OTUs. Ideas derived from complementarity have also been exploited by trait-based modeling approaches (54). However, neither complementarity nor compensatory dynamics explains the observed succession in OTUs during phase IV of our experiment, where external drivers were constant.

Theories such as niche complementarity, niche construction, resource partitioning, cross-feeding, and others have been proposed to explain internal dynamics that occur in the absence of external forcing (40). A basic premise in these theories is that organisms modify their environment, thereby creating new niches that can be exploited by others, which can lead to a natural and perpetuating succession of organisms that can occur rapidly in microbial systems (55–57). One type of niche creation is known as cross-feeding, in which the waste products of one organism’s metabolism become the food for another. In the original study by Rosenzweig et al. (58), cross-feeding developed from a clonal population of *Escherichia coli* that oxidized glucose completely to CO_2_, but after more than 700 generations, stable polymorphisms evolved that produced and consumed acetate and glycerol intermediates (see also reference 59). Hence, the clonal population naturally evolved a type of distributed metabolism (60). Syntrophy and resource partitioning are also examples of cross-feeding that develop between species, phyla, and domains (61, 62), where differential production of shared intermediates over time can give rise to asymmetric population dynamics that can stabilize ecosystem function (63, 64). One mechanism that may drive evolution of cross-feeding is the interplay between growth rate and growth efficiency.

Metabolic analysis in substrate-limited systems has shown that metabolic pathway truncation, such as partial oxidation of glucose to acetate instead of CO_2_, can result in faster energy extraction per unit of time, which can support higher growth rates but leads to the excretion of by-products, such as acetate (65, 66). The accumulation of intermediates can then foster adaptive gene loss that reinforces cross-feeding (67, 68). Leaking substrates is in contrast to conventional wisdom that considers maximizing the efficiency of substrate use a virtue. However, a recent modeling study by González-Cabaleiro et al. (69) examined these tradeoffs and showed that maximizing the rate of energy harvest from substrates, with its attendant by-product production, accurately predicts the distribution of metabolic labor observed in multistep anaerobic fermentation of glucose to methane and CO_2_, two-step aerobic autotrophic nitrification, and single-step anaerobic denitrification. Furthermore, excreted substrates in communities can drive the production of new metabolites that are otherwise not produced when organisms are grown in isolation, via emergent biosynthetic capacity (70). Consequently, we speculate that a potential driver of the rapid succession of dominant OTUs observed in both the control and cycled chemostats may be continuous niche reconstruction via the extracellular accumulation of metabolic intermediates. As intermediate metabolites accumulate beyond certain thresholds, select members of the rare biosphere may be freed from dormancy by competitive advantages that allow them to achieve dominance, but only temporarily. New dominant OTUs might excrete new intermediate metabolites that then eventually select for new replacements. With sufficient biodiversity, intermediate metabolites come and go, but none accumulate significantly, so ecosystem functions like the methane oxidation rate or primary production proceed at maximum despite the continuous species turnover. Of course, in some situations, violent perturbations can lead to excessive accumulation of metabolites and cause system collapse (71).

Dynamic cross-feeding is not the only process shaping communities. Depending on the characteristic time scales of internal and external forcing, generalists and specialists also arise (72) and various types of chemical warfare are likely at play (73). Cooperation via cross-feeding (74, 75), quorum sensing (76), stigmergy (77), horizontal gene transfer (78), and other types of intercellular communication (79, 80) also contribute to internal feedbacks that likely support the continuous succession of dominant OTUs we observed. Indeed, the continual turnover of the community may be a significant mechanism in producing and maintaining the rank abundance distribution of the rare biosphere (44). Furthermore, the internal dynamics exhibited by microbial systems brings into question the usefulness of stability criteria often used to assess and cull food web models. If microbial community dynamics are fundamentally unstable (81), then predicting ecosystem function based on maximizing dissipation of energy may be a more tractable approach for understanding how communities will change in response to external forcing (23).

Our results, as well as results from a previous modeling study (26), indicate that the microbial communities in our methanotrophic microcosms are inherently well adapted to periodic inputs of energy, likely due to the implementation of temporal strategies like resource storage. The 16S rRNA gene sequences were sampled deeply at 10 time points during the 510-day experiment and show that the dominant OTU at any time point often originated from the rare biosphere but was subsequently replaced by a new competitor also derived from the rare biosphere at the next sample point. Even though the control and cycled chemostats experienced significantly different external forcing and the overall community composition changed as the experiment proceeded, the patterns of succession of dominant OTUs in both treatments were more similar than different. These results indicate that internal feedbacks were more important than external drivers in shaping the community dynamics over time. Based on supportive data in the literature, we speculate that dynamic cross-feeding may be the mechanism producing the unstable community dynamics. Furthermore, our results, as well as those of others, bring into question the usefulness of the concept of stability for understanding microbial food webs. Because the ecosystem function measured here, the methane oxidation rate, was insensitive to the community dynamics, our results support the conjectures that microbial system organization results in a state that maximizes energy dissipation and that many different food web configurations can support the same function, as evidenced by the observed community succession but nearly constant methane oxidation rate.

## MATERIALS AND METHODS

### Experiment setup and sampling.

**FIG 6:**
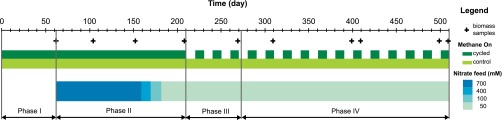
Experiment timeline showing the different phases of the study, the presence or absence of methane in the gas feed for each treatment, the changes in nitrate concentrations in the liquid feed, and the sampling dates for microbial community characterization. Phase I, batch mode; phase II, start-up; phase III, cycling; phase IV, pH-controlled cycling.

The experimental set up consisted of four 18-liter Bellco Glass bioreactors housed in a dark Conviron environmental chamber controlled at 20°C. The microcosms were previously inoculated with 1 liter of unfiltered water collected from a cedar bog in Falmouth, MA, and sparged with a gas mix containing 4.9% methane in air at a gas flow rate of 20 ml ⋅ min^−1^ (for details see Text S1 in the supplemental material). The experiment consisted of four phases (Fig. 6). In phase I (days 0 to 62), the microcosms initially operated in batch mode, but all reactors were interconnected in a closed loop at a flow rate of 10 ml ⋅ min^−1^ to ensure uniform community composition between chemostats. In phase II (days 63 to 209), the microcosms operated independently in chemostat mode with a defined mineral salt medium (70 µM K_2_HPO_4_, 700 µM KNO_3_, 100 µM MgSO_4_, 100 µM CaCl_2_, 100 µM NaCl) plus trace elements (final concentrations, 18.50 µM FeCl_3_ 6H_2_O, 0.49 µM H_3_BO_3_, 0.13 µM CoCl_2_ 6H_2_O, 0.10 µM CuSO_4_ 5H_2_O, 0.35 µM ZnSO_4_ 7H_2_O, 0.16 µM MnSO_4_ H_2_O, 0.12 µM Na_2_MoO_4_ 2H_2_O, 0.08 µM NiCl_2_ 6H_2_O, and 0.1 mM HCl) at dilution rate of 0.1 day^−1^ (1.25 ml ⋅ min^−1^). The nitrate concentration was adjusted decrementally from 700 µM to 50 µM to ensure N-limited rather than CH_4_-limited growth. In phase III (days 210 to 273), duplicate chemostats were divided into control and cycled treatments. The cycled chemostats were subjected to periodic energy input cycles by switching the gas composition from a methane (4.9%)-plus-air mixture to solely air over a 20-day period (i.e., 10 days CH_4_-on, 10 days CH_4_-off). The two control chemostats were maintained under a continuous input of 4.9% methane in air (Fig. 6). In phase IV (days 274 to 510), gas cycling continued but passive pH control was initiated by adding 10 mM potassium phosphate buffer to the feed medium. Liquid samples were withdrawn periodically for analysis of nitrate plus nitrite (NO_3_^−^), ammonia (NH4^+^), particulate organic carbon (POC) and nitrogen (PON), dissolved organic carbon (DOC) and nitrogen (DON), and microbial cell abundances (both eukaryotic and prokaryotic organisms). The CH_4_, O_2_, and CO_2_ gas concentrations in the feed and headspace were automatically measured and recorded every 5 h, and the pH was recorded every hour. For details on the analysis, see the supplemental material. Biomass samples for 16S rRNA gene sequencing were taken on 10 different days, where all samples except those from days 399 and 499 corresponded to periods when CH_4_ was on in the cycled chemostats (Fig. 6). The chemostats were thoroughly resuspended via mixing before filtering; the resulting homogenized supernatant was filtered through 0.22-µm Sterivex-GP membranes. Our target for filtration was a 600-ml volume or until the filter clogged. Filters were immediately frozen at −80°C until DNA extractions.

10.1128/mSystems.00117-16.1 Text S1  Detailed description of the experiment setup, determination of microbial cell abundances, and chemical and statistical analyses. Download Text S1, DOCX file, 0.1 MB. Copyright © 2016 Fernandez-Gonzalez et al. 2016 Fernandez-Gonzalez et al. This content is distributed under the terms of the Creative Commons Attribution 4.0 International license.

### DNA extraction, pyrosequencing, and sequence analysis.

Total genomic DNA was extracted from whole Sterivex filters that were thawed and cut into small pieces of similar size using the Sterivex internal support as a guide, prior to the extraction of nucleic acids using the RNA PowerSoil total RNA isolation kit, combined with the DNA elution accessory kit (MoBio, Carlsbad, CA), following the manufacturer’s protocol. The DNA concentrations were determined with the Quant-iT PicoGreen double-stranded DNA (dsDNA) assay kit (Life Technologies, Grand Island, NY, USA). Amplicon libraries for the V4-V6 region of 16S rRNA bacterial genes were prepared using fused primers and sequenced using Roche Titanium technology as previously described (82). Three technical PCR replicates per DNA extraction were performed, with 5 to 20 ng of DNA per PCR. The sequencing reads were quality filtered to remove any reads that contained ambiguous bases, had average quality scores below Q30, or lacked exact primer matches. Quality-filtered sequences were analyzed for chimera removal with UCHIME (83), combining both *de novo* and reference database (ChimeraSlayer Gold) modes, and then clustered at 0.96 similarity with UCLUST (84) to define OTUs. Taxonomy was assigned by global alignment for sequence taxonomy (GAST [85]) with the SILVA database (86). Quality-filtered sequences are publicly available through the VAMPS database (https://vamps.mbl.edu) under project number JAH_ENT_Bv6v4.

Statistical analysis of OTU abundances was performed with QIIME 1.8 (87), Primer 6, PERMANOVA+ (Primer-E Ltd., Plymouth, United Kingdom) (88), and R (89). To compare bacterial communities and estimate community turnover, a distance matrix was calculated using the Morisita-Horn dissimilarity index (MH) (90) of log-transformed rarefied data. Nonmetric multidimensional scaling (NMDS) analysis was applied to explore distances among communities. Differences between treatments (control and cycled) and time (sampling day) were tested with PERMANOVA tests (91) with 1,000 replications, including pairwise comparisons between individual samples. Additional analyses are described in Text S1 in the supplemental material.

### Accession number(s).

All sequences produced in this study are available in the NCBI Short Read Archive under accession number PRJNA322031.
