# Supplementary material for: Microbial Communities Are Well Adapted to Disturbances in Energy Input
Source: mSystems. 2016 Sep 13;1(5):e00117-16. doi: 10.1128/mSystems.00117-16 (PMC5080406; doi:10.1128/mSystems.00117-16)
Supplement: Table S1 [file sys005162053st2.docx]

**Table S1A.** Number of sequences, observed OTUs, Good’s library coverage and Simpson diversity and Evenness indices for each of the ten samples from each microcosm.

|  |  | **Cycled 1** | | | | |  |  | **Cycled 2** | | | | |  |
| --- | --- | --- | --- | --- | --- | --- | --- | --- | --- | --- | --- | --- | --- | --- |
| **time (d)** | **Seq^a^** | | **OTU** | **G^b^** | **1-D** | **E** | | **Seq** | | **OTU** | **G** | **1-D** | **E** | |
| 62 | 10731 | | 1188 | 0.65 | 0.88 | 1.2×10^-3^ | | 13103 | | 1391 | 0.66 | 0.93 | 0.0011 | |
| 104 | 12405 | | 1308 | 0.70 | 0.94 | 1.1×10^-3^ | | 12413 | | 1072 | 0.59 | 0.91 | 0.0014 | |
| 152 | 14966 | | 1132 | 0.83 | 0.97 | 1.3×10^-3^ | | 17075 | | 1994 | 0.58 | 0.92 | 0.0010 | |
| 208 | 13444 | | 1091 | 0.76 | 0.85 | 1.5×10^-3^ | | 13857 | | 806 | 0.57 | 0.71 | 0.0025 | |
| 269 | 11759 | | 326 | 0.96 | 0.58 | 6.0×10^-3^ | | 14662 | | 334 | 0.94 | 0.53 | 0.0069 | |
| 309 | 10160 | | 200 | 0.96 | 0.90 | 6.0×10^-3^ | | 16378 | | 293 | 0.68 | 0.85 | 0.0053 | |
| 399 | 11748 | | 1167 | 0.62 | 0.97 | 1.2×10^-3^ | | 11831 | | 592 | 0.69 | 0.85 | 0.0025 | |
| 409 | 12836 | | 953 | 0.67 | 0.74 | 2.0×10^-3^ | | 12670 | | 701 | 0.71 | 0.84 | 0.0023 | |
| 499 | 13200 | | 2142 | 0.44 | 0.98 | 7×10^-4^ | | 14519 | | 547 | 0.97 | 0.95 | 0.0025 | |
| 509 | 10307 | | 1071 | 0.66 | 0.95 | 1.2×10^-3^ | | 11539 | | 949 | 0.94 | 0.95 | 0.0014 | |
| ***Total*** | *121556* | | *6801* |  |  |  | | *138047* | | *5612* |  |  |  | |
| ***avg*** |  | |  | *0.72* |  |  | |  | |  | *0.76* |  |  | |
|  |  | **Control 1** | | | | |  |  | **Control 2** | | | | |  |
| **time (d)** | **Seq** | | **OTU** | **G** | **1-D** | **E** | | **Seq** | | **OTU** | **G** | **1-D** | **E** | |
| 62 | 12114 | | 1189 | 0.70 | 0.88 | 0.0013 | | 13150 | | 1109 | 0.63 | 0.90 | 0.0014 | |
| 104 | 12267 | | 1712 | 0.70 | 0.91 | 0.0009 | | 13131 | | 1043 | 0.70 | 0.74 | 0.0018 | |
| 152 | 15234 | | 2440 | 0.52 | 0.98 | 0.0007 | | 14751 | | 2455 | 0.57 | 0.94 | 0.0007 | |
| 208 | 11825 | | 1876 | 0.92 | 0.98 | 0.0007 | | 12499 | | 594 | 0.76 | 0.76 | 0.0027 | |
| 269 | 10574 | | 347 | 0.94 | 0.81 | 0.0039 | | 8888 | | 169 | 0.96 | 0.82 | 0.0074 | |
| 309 | 14695 | | 780 | 0.92 | 0.90 | 0.0021 | | 11606 | | 173 | 0.95 | 0.83 | 0.0078 | |
| 399 | 11353 | | 506 | 0.74 | 0.76 | 0.0032 | | 17288 | | 450 | 0.79 | 0.34 | 0.0109 | |
| 409 | 12798 | | 671 | 0.70 | 0.89 | 0.0023 | | 9743 | | 747 | 0.68 | 0.87 | 0.0018 | |
| 499 | 14340 | | 404 | 0.49 | 0.97 | 0.0030 | | 13633 | | 1809 | 0.92 | 0.98 | 0.0009 | |
| 509 | 7984 | | 411 | 0.83 | 0.94 | 0.0026 | | 14153 | | 847 | 0.65 | 0.96 | 0.0016 | |
| ***Total*** | *123184* | | *6824* |  |  |  | | *128842* | | *6532* |  |  |  | |
| ***avg*** |  | |  | *0.73* |  |  | |  | |  | *0.75* |  |  | |

^a^ sequences

^b^ Good´s library coverage

**Table S1B.** PERMANOVA pairwise tests for bacterial community dissimilarities at different sampling times during Phase II (one way PERMANOVA, factor: time) and Phase III and IV (two-way PERMANOVA, factors: treatment and time). Monte-Carlo P values were calculated after 9999 permutations.

| **Days** | **t** | **P** |
| --- | --- | --- |
| **Phase II** |  |  |
| 62, 104 | 1.69 | 0.1185 |
| 62, 152 | 9.3259 | 0.0001** |
| 62, 208 | 5.0751 | 0.0004** |
| 104, 152 | 5.8231 | 0.001** |
| 152, 208 | 4.2431 | 0.0014** |
| 104, 208 | 4.4233 | 0.0009** |
| **Phase III and IV** |  |  |
| 269, 309 | 3.4515 | 0.0078** |
| 269, 399 | 2.6204 | 0.0157 |
| 269, 409 | 3.3978 | 0.0083** |
| 269, 499 | 3.8742 | 0.0045** |
| 269, 509 | 3.1221 | 0.0068** |
| 309, 399 | 1.5253 | 0.1398 |
| 309, 409 | 1.3589 | 0.2267 |
| 309, 499 | 1.9736 | 0.05 |
| 309, 509 | 1.3901 | 0.197 |
| 399, 409 | 0.61594 | 0.7299 |
| 399, 499 | 1.4133 | 0.1662 |
| 399, 509 | 0.92122 | 0.4692 |
| 409, 499 | 1.9288 | 0.0487 |
| 409, 509 | 0.92034 | 0.4541 |
| 499, 509 | 0. 82029 | 0. 5312 |
| ** (p-values < 0.01) |  |  |
