# Supplementary material for: Microbial Communities Are Well Adapted to Disturbances in Energy Input
Source: mSystems. 2016 Sep 13;1(5):e00117-16. doi: 10.1128/mSystems.00117-16 (PMC5080406; doi:10.1128/mSystems.00117-16)
Supplement: Table S2 [file sys005162053st3.pdf]

Table S2. Relative abundances lower taxonomic groups that contains abundant OTUs averaged by experimental phases.

**TAXONOMIC GROUPS**				**RELATIVE ABUNDANCES (%)**															
**Domain**				**Cycled 1**				**Cycled 2**				**Control 1**				**Control 2**			
**Phylum**																			
**Class**	**Order**	**Family**	**Genera**	**I**	**II**	**III**	**IV**	**I**	**II**	**III**	**IV**	**I**	**II**	**III**	**IV**	**I**	**II**	**III**	**IV**
Bacteria																			
Acidobacteria																			
Acidobacteria	*Acidobacteriales*	*Acidobacteriaceae*	*Chloroacidobacterium*	7.56	5.57	0.03	0.00	6.26	0.79	0.00	0.01	6.32	9.74	0.40	0.02	8.06	2.62	0.00	0.11
Acidobacteria	*Acidobacteriales*	*Acidobacteriaceae*	Other	5.93	2.71	0.38	3.51	7.35	1.56	0.27	2.81	6.54	2.51	0.39	0.54	5.04	1.45	0.00	2.89
Holophagae	Other	Other	Other	0.00	0.03	0.01	1.05	0.03	0.00	0.00	0.00	0.00	0.02	0.00	0.00	0.00	0.00	0.00	0.00
Actinobacteria																			
Actinobacteria	*Actinomycetales*	*Mycobacteriaceae*	*Mycobacterium*	0.03	0.47	0.77	1.67	0.06	0.70	0.46	0.73	0.03	0.26	0.71	0.59	0.17	0.41	0.56	0.87
Actinobacteria	*Actinomycetales*	*Nocardiaceae*	*Rhodococcus*	0.00	0.00	0.03	0.12	0.00	0.00	0.03	0.04	0.00	0.01	0.00	0.02	0.00	0.00	1.07	0.04
Bacteroidetes																			
Flavobacteria	*Flavobacteriales*	*Flavobacteriaceae*	*Flavobacterium*	0.00	0.38	0.00	0.04	0.00	0.16	0.00	0.02	0.00	0.17	0.03	0.23	0.01	0.64	0.00	0.09
Sphingobacteria	*Sphingobacteriales*	*Chitinophagaceae*	*Ferruginibacter*	0.23	0.93	0.03	0.09	0.19	0.45	0.02	4.89	0.30	1.11	0.08	0.24	0.29	0.54	0.00	0.96
Sphingobacteria	*Sphingobacteriales*	*Chitinophagaceae*	*Flavisolibacter*	0.40	0.72	0.07	0.00	0.28	0.26	0.00	0.00	0.38	0.87	0.37	0.00	0.40	0.21	0.00	0.00
Sphingobacteria	*Sphingobacteriales*	*Chitinophagaceae*	Other	2.81	3.00	0.48	1.11	2.24	1.32	0.25	0.48	2.23	4.97	0.49	0.87	3.25	2.13	0.89	1.46
Sphingobacteria	*Sphingobacteriales*	*Chitinophagaceae*	*Sediminibacterium*	0.02	0.36	0.12	1.24	0.08	0.71	0.18	0.65	0.00	0.17	0.38	2.22	0.03	0.43	1.71	0.45
Sphingobacteria	*Sphingobacteriales*	*Chitinophagaceae*	*Terrimonas*	0.16	0.09	0.01	0.19	0.10	0.04	0.01	0.08	0.12	0.17	0.03	0.01	0.25	0.12	0.00	0.49
Sphingobacteria	*Sphingobacteriales*	*Cytophagaceae*	*Flexibacter*	0.20	2.84	2.21	2.31	0.24	3.81	3.44	2.05	0.18	2.01	0.46	0.90	0.19	2.59	2.33	2.52
Sphingobacteria	*Sphingobacteriales*	Other	Other	0.56	1.86	0.11	2.07	0.55	2.16	0.04	0.31	0.56	3.44	0.74	0.64	0.73	3.76	0.02	0.99
Sphingobacteria	*Sphingobacteriales*	*Saprospiraceae*	*Haliscomenobacter*	0.04	0.15	0.00	0.00	0.02	0.15	0.00	0.00	0.03	0.23	0.00	0.04	0.02	0.15	0.00	0.27
Sphingobacteria	*Sphingobacteriales*	*Saprospiraceae*	*Lewinella*	1.60	0.38	0.00	0.00	0.98	0.03	0.00	0.00	1.39	0.28	0.00	0.00	1.74	0.08	0.00	0.00
Sphingobacteria	*Sphingobacteriales*	*Saprospiraceae*	Other	0.00	0.19	0.01	0.00	0.01	0.78	0.00	0.00	0.01	0.07	0.00	0.00	0.00	0.26	0.00	0.74
Sphingobacteria	*Sphingobacteriales*	*Sphingobacteriaceae*	*Sphingobacteriaceae*	0.00	0.03	0.06	0.00	0.00	0.01	0.00	0.01	0.01	0.02	3.03	0.00	0.01	0.02	0.00	0.00
Chlamydiae																			
Chlamydiae	*Chlamydiales*	Other	Other	0.05	0.05	0.00	0.23	0.12	0.06	0.00	0.02	0.12	0.11	0.10	4.60	0.09	0.01	0.00	0.09
Chlamydiae	*Chlamydiales*	*Parachlamydiaceae*	*Neochlamydia*	0.00	0.04	0.02	1.70	0.02	0.07	0.00	1.18	0.02	0.10	0.01	0.32	0.03	0.03	0.00	0.09
Chlamydiae	*Chlamydiales*	*Parachlamydiaceae*	*Protochlamydia*	0.01	0.16	0.02	0.15	0.01	0.14	0.07	0.09	0.02	0.29	0.02	0.34	0.01	0.10	0.00	0.26
Chlamydiae	*Chlamydiales*	*Simkaniaceae*	*Rhabdochlamydia*	0.14	0.12	0.00	0.21	0.31	0.12	0.05	0.05	0.31	0.40	0.18	0.65	0.71	0.07	0.00	0.15
Chlorobi																			
Chlorobia	*Chlorobiales*	Other	Other	0.07	1.33	0.56	0.22	0.08	0.89	0.27	0.16	0.10	0.53	0.16	0.18	0.05	0.51	0.01	0.32
Chloroflexi																			
Anaerolineae	*Anaerolineales*	*Anaerolinaceae*	Other	0.07	0.29	0.20	1.59	0.05	0.08	0.04	0.33	0.11	0.28	0.04	0.17	0.05	0.09	0.21	0.45
Caldilineae	*Caldilineales*	*Caldilineaceae*	*Caldilinea*	0.12	1.42	0.53	2.60	0.23	1.01	0.70	1.38	0.12	1.56	0.58	0.20	0.15	1.18	1.47	1.71
Caldilineae	*Caldilineales*	Other	Other	0.01	0.30	0.14	1.44	0.02	0.34	0.13	1.84	0.01	0.32	0.07	0.51	0.02	0.58	0.01	1.86
Cyanobacteria																			
Other	Other	Other	Other	3.84	2.49	0.70	1.80	4.93	0.96	0.36	1.05	5.67	2.82	0.30	0.85	5.05	1.12	3.93	0.85
Firmicutes																			
Bacilli	*Bacillales*	*Paenibacillaceae*	*Paenibacillus*	0.00	0.04	0.00	0.05	0.00	0.04	0.00	0.01	0.00	0.02	0.01	0.82	0.01	0.02	0.00	0.46
Nitrospirae																			
Nitrospira	*Nitrospirales*	*Nitrospiraceae*	*Nitrospira*	0.13	0.18	0.00	0.36	0.13	0.01	0.00	0.13	0.09	0.21	0.00	0.03	0.09	0.09	0.00	0.13
OD1																			
Other	Other	Other	Other	0.00	0.14	0.03	0.65	0.04	0.13	0.14	0.03	0.00	0.11	0.00	0.46	0.00	0.00	0.17	0.21
OP10																			
Other	Other	Other	Other	0.25	0.33	0.09	0.54	0.35	0.25	0.01	0.34	0.37	0.43	0.06	0.42	0.31	0.23	0.00	0.64
Planctomycetes																			
Other	Other	Other	Other	0.00	2.53	0.00	0.03	0.00	0.28	0.02	0.45	0.00	1.20	0.50	0.05	0.01	0.45	0.01	0.06
Phycisphaerae	Other	Other	Other	0.24	0.33	0.03	0.06	0.27	0.22	0.15	0.09	0.14	0.28	0.41	0.63	0.12	0.13	0.00	0.55
Planctomycetacia	*Planctomycetales*	*Planctomycetaceae*	*Gemmata*	0.31	0.32	0.06	0.04	0.45	0.20	0.03	0.04	0.19	0.98	0.70	0.06	0.24	0.35	0.09	0.10
Planctomycetacia	*Planctomycetales*	*Planctomycetaceae*	Other	1.36	0.64	0.08	0.71	2.27	0.38	0.09	0.31	1.24	1.20	0.54	0.24	1.07	0.31	0.05	0.41
Proteobacteria																			
Alphaproteobacteria	*Caulobacterales*	*Caulobacteraceae*	*Asticcacaulis*	0.00	0.05	0.00	0.00	0.00	0.69	0.05	0.00	0.04	0.09	0.53	0.11	0.02	0.28	1.06	0.00
Alphaproteobacteria	*Caulobacterales*	*Hyphomonadaceae*	Other	0.09	0.19	0.14	0.15	0.12	0.17	0.12	0.21	0.07	0.22	0.12	0.25	0.09	0.11	1.23	0.67
Alphaproteobacteria	*Rhizobiales*	*Beijerinckiaceae*	Other	1.65	1.47	0.91	7.34	5.36	1.79	1.13	3.99	1.84	1.64	6.75	7.54	2.58	1.03	1.05	4.84
Alphaproteobacteria	*Rhizobiales*	*Bradyrhizobiaceae*	*Bradyrhizobium*	0.08	0.12	0.41	0.29	0.20	0.06	0.12	0.03	0.07	0.20	0.03	0.02	0.11	0.11	4.82	0.12
Alphaproteobacteria	*Rhizobiales*	*Bradyrhizobiaceae*	*Rhodopseudomonas*	0.62	0.02	0.00	0.00	1.27	0.04	0.00	0.00	0.45	0.02	0.00	0.00	0.62	0.02	1.15	0.00
Alphaproteobacteria	*Rhizobiales*	*Hyphomicrobiaceae*	*Ancylobacter*	0.00	0.00	0.00	0.00	0.00	0.00	0.00	0.00	0.00	0.00	0.00	6.32	0.00	0.00	0.00	0.47
Alphaproteobacteria	*Rhizobiales*	*Hyphomicrobiaceae*	*Hyphomicrobium*	0.08	1.20	0.14	0.35	0.23	1.93	0.05	0.44	0.08	0.97	0.91	1.00	0.11	1.32	0.87	0.34
Alphaproteobacteria	*Rhizobiales*	*Methylocystaceae*	Other	0.20	0.84	0.43	0.87	0.24	0.40	0.16	0.36	0.21	0.92	0.90	0.21	0.15	0.46	0.59	0.22
Alphaproteobacteria	*Rhodospirillales*	Other	Other	0.28	0.48	0.22	1.27	0.50	0.34	0.19	0.50	0.31	0.55	0.71	0.44	0.33	0.21	0.06	0.48
Alphaproteobacteria	*Rhodospirillales*	*Rhodospirillaceae*	Other	0.24	0.66	0.06	0.34	0.40	0.58	0.27	0.44	0.19	0.52	0.29	1.11	0.30	0.31	0.00	0.21
Alphaproteobacteria	*Rickettsiales*	Other	Other	0.05	1.52	0.14	0.23	0.06	0.11	0.10	0.12	0.04	0.28	0.14	0.99	0.03	1.08	0.00	0.21
Alphaproteobacteria	*Rickettsiales*	Unassigned	*Caedibacter*	0.00	0.00	0.00	0.07	0.00	0.00	0.00	0.43	0.00	0.00	0.00	0.14	0.00	0.00	0.00	0.35
Alphaproteobacteria	*Sphingomonadales*	*Sphingomonadaceae*	Other	1.36	0.26	0.00	0.00	1.91	0.12	0.00	0.00	1.98	0.12	0.00	0.00	1.43	0.24	0.00	0.00
Alphaproteobacteria	*Sphingomonadales*	*Sphingomonadaceae*	*Sphingomonas*	4.84	0.50	0.09	0.08	8.75	0.24	0.08	0.04	5.23	0.83	0.09	0.04	5.08	0.32	0.48	0.00
Betaproteobacteria	*Burkholderiales*	*Burkholderiaceae*	*Limnobacter*	0.01	0.37	0.12	0.00	0.00	0.00	0.10	0.00	0.00	0.01	0.00	0.00	0.00	0.00	0.52	0.00
Betaproteobacteria	*Burkholderiales*	*Burkholderiaceae*	*Ralstonia*	0.00	0.01	0.37	0.39	0.00	0.00	0.20	0.03	0.02	0.01	0.26	0.03	0.05	0.00	9.06	0.25
Betaproteobacteria	*Burkholderiales*	*Comamonadaceae*	*Methylibium*	0.16	0.23	0.30	0.12	0.15	0.19	0.05	0.51	0.07	0.22	0.00	0.14	0.07	0.26	0.54	0.09
Betaproteobacteria	*Burkholderiales*	*Comamonadaceae*	Other	0.08	0.66	0.82	0.47	0.23	0.76	3.79	0.75	0.08	0.75	0.58	0.91	3.17	0.63	1.96	0.80
Betaproteobacteria	*Burkholderiales*	*Oxalobacteraceae*	*Janthinobacterium*	0.00	0.34	0.00	0.04	0.00	0.49	0.00	0.09	0.00	0.11	0.06	0.01	0.01	0.14	0.00	0.01
Betaproteobacteria	*Burkholderiales*	*Oxalobacteraceae*	Other	0.00	0.03	0.00	0.00	0.00	0.03	0.00	0.00	0.00	0.04	3.04	0.00	0.00	0.03	0.00	0.00
Betaproteobacteria	*Methylophilales*	*Methylophilaceae*	*Methylobacillus*	0.00	1.51	0.00	0.00	0.01	0.47	0.80	0.01	0.00	0.75	0.40	0.06	0.01	0.79	1.77	0.18
Betaproteobacteria	*Methylophilales*	*Methylophilaceae*	*Methylophilus*	0.03	7.77	9.72	9.13	0.03	8.32	8.29	20.53	0.05	7.47	7.15	1.13	0.05	11.15	1.99	11.46
Betaproteobacteria	*Methylophilales*	*Methylophilaceae*	Other	0.00	0.01	0.15	0.01	0.00	0.29	0.10	0.01	0.02	0.14	0.57	3.63	0.01	0.09	1.01	2.03
Betaproteobacteria	*Neisseriales*	*Neisseriaceae*	Other	0.59	0.05	0.03	0.65	0.85	0.03	0.16	0.42	0.88	0.24	0.00	1.68	1.13	0.03	0.00	1.35
Betaproteobacteria	*Nitrosomonadales*	*Nitrosomonadaceae*	Other	0.45	0.96	0.37	0.33	0.34	0.68	0.06	0.14	0.35	1.59	0.44	0.03	0.14	0.58	0.00	0.12
Deltaproteobacteria	*Bdellovibrionales*	*Bdellovibrionaceae*	*Bdellovibrio*	0.07	1.02	0.20	0.22	0.05	0.59	0.03	0.11	0.04	0.67	0.04	0.05	0.05	0.82	0.00	0.02
Deltaproteobacteria	*Myxococcales*	*Haliangiaceae*	*Haliangium*	0.21	1.19	0.17	0.04	0.15	0.56	0.19	0.05	0.17	0.72	0.27	0.07	0.20	1.20	0.45	0.14
Deltaproteobacteria	*Myxococcales*	Other	Other	0.19	0.74	0.19	0.72	0.11	0.70	0.44	2.45	0.12	0.65	0.13	0.05	0.24	0.33	0.12	0.79
Gammaproteobacteria	*Enterobacteriales*	*Enterobacteriaceae*	Other	0.00	0.02	0.71	0.32	0.01	0.00	0.21	0.03	0.00	0.00	0.60	0.13	0.00	0.03	3.67	0.55
Gammaproteobacteria	*Legionellales*	*Coxiellaceae*	*Aquicella*	0.10	0.20	0.13	1.08	0.03	0.06	0.04	14.74	0.09	0.22	0.33	27.07	0.06	0.07	0.00	10.31
Gammaproteobacteria	*Legionellales*	*Coxiellaceae*	*Rickettsiella*	0.01	0.01	0.00	0.96	0.01	0.00	0.00	0.03	0.01	0.00	0.00	0.27	0.00	0.00	0.00	1.04
Gammaproteobacteria	*Legionellales*	*Legionellaceae*	*Legionella*	0.10	0.19	0.00	0.32	0.12	0.40	0.03	0.37	0.08	0.37	0.00	0.69	0.13	0.20	0.00	0.11
Gammaproteobacteria	*Methylococcales*	*Crenotrichaceae*	Other	0.01	0.05	0.11	4.32	0.00	0.04	0.06	3.06	0.02	0.06	0.00	0.70	0.00	0.02	0.00	2.53
Gammaproteobacteria	*Methylococcales*	*Methylococcaceae*	*Methylobacter*	0.05	0.05	0.01	25.10	0.06	0.54	0.18	13.04	0.07	1.62	11.56	10.72	0.03	0.22	0.00	29.06
Gammaproteobacteria	*Methylococcales*	*Methylococcaceae*	*Methylomonas*	32.21	16.33	0.92	3.88	23.12	22.06	0.27	7.22	33.04	18.43	0.67	2.69	29.77	26.30	0.00	1.22
Gammaproteobacteria	*Methylococcales*	*Methylococcaceae*	*Methylosoma*	0.28	14.08	68.03	0.27	0.15	24.82	70.97	0.58	0.25	4.13	43.68	0.06	0.17	17.16	42.92	0.09
Gammaproteobacteria	*Methylococcales*	*Methylococcaceae*	Other	1.95	0.59	0.00	0.03	1.63	0.47	0.00	0.02	2.18	1.06	0.02	0.00	1.73	1.04	0.00	0.02
Gammaproteobacteria	*Methylococcales*	Other	Other	0.07	1.59	0.20	0.07	0.11	0.83	0.09	0.03	0.06	0.38	0.03	0.00	0.09	2.26	0.00	0.03
Gammaproteobacteria	Other	Other	Other	0.35	1.18	0.18	0.94	0.35	0.47	0.15	1.22	0.17	0.82	0.08	5.23	0.30	0.76	0.44	0.61
Gammaproteobacteria	*Pseudomonadales*	*Moraxellaceae*	*Acinetobacter*	0.00	0.00	0.00	0.29	0.00	0.00	0.00	0.00	0.00	0.00	0.04	0.00	0.00	0.01	0.00	0.00
Gammaproteobacteria	*Xanthomonadales*	*Sinobacteraceae*	*Nevskia*	18.30	0.85	0.04	0.00	12.79	0.80	0.05	0.02	15.87	1.08	0.00	0.01	13.19	0.50	0.00	0.00
Gammaproteobacteria	*Xanthomonadales*	*Sinobacteraceae*	Other	0.24	0.32	0.31	0.13	0.24	0.21	0.47	0.12	0.28	0.34	0.54	0.81	0.25	0.42	1.15	0.29
Verrucomicrobia																			
Opitutae	*Opitutales*	*Opitutaceae*	*Opitutus*	0.07	0.76	0.47	0.63	0.02	0.92	0.42	0.54	0.04	0.55	0.60	0.09	0.04	0.49	0.35	0.22
Spartobacteria	*Chthoniobacter*	Other	Other	0.09	0.19	2.04	0.06	0.16	0.30	0.83	0.03	0.14	0.12	0.17	0.01	0.08	0.09	2.05	0.14
Spartobacteria	*Chthoniobacterales*	*Xiphinematobacteriaceae*	Other	0.00	0.40	0.37	0.37	0.00	1.15	0.18	0.29	0.00	0.99	0.51	1.34	0.01	0.15	0.19	1.78
Spartobacteria	Other	Other	Other	0.13	1.14	0.04	3.64	0.21	1.87	0.02	1.75	0.18	1.60	0.09	2.86	0.17	1.84	0.00	0.64
Verrucomicrobiae	*Verrucomicrobiales*	Subdivision3	Other	0.23	1.05	0.31	2.13	0.36	0.47	0.31	1.02	0.35	0.98	0.26	0.23	0.18	0.48	0.32	0.47
Unknown																			
Other																			
Other	Other	Other	Other	0.89	0.61	0.07	0.75	1.11	0.38	0.13	0.56	0.69	0.75	0.28	1.28	0.85	0.48	0.61	1.17
