# Supplementary figures and images for: Microbial Communities Are Well Adapted to Disturbances in Energy Input
Source: mSystems. 2016 Sep 13;1(5):e00117-16. doi: 10.1128/mSystems.00117-16 (PMC5080406; doi:10.1128/mSystems.00117-16)

a)

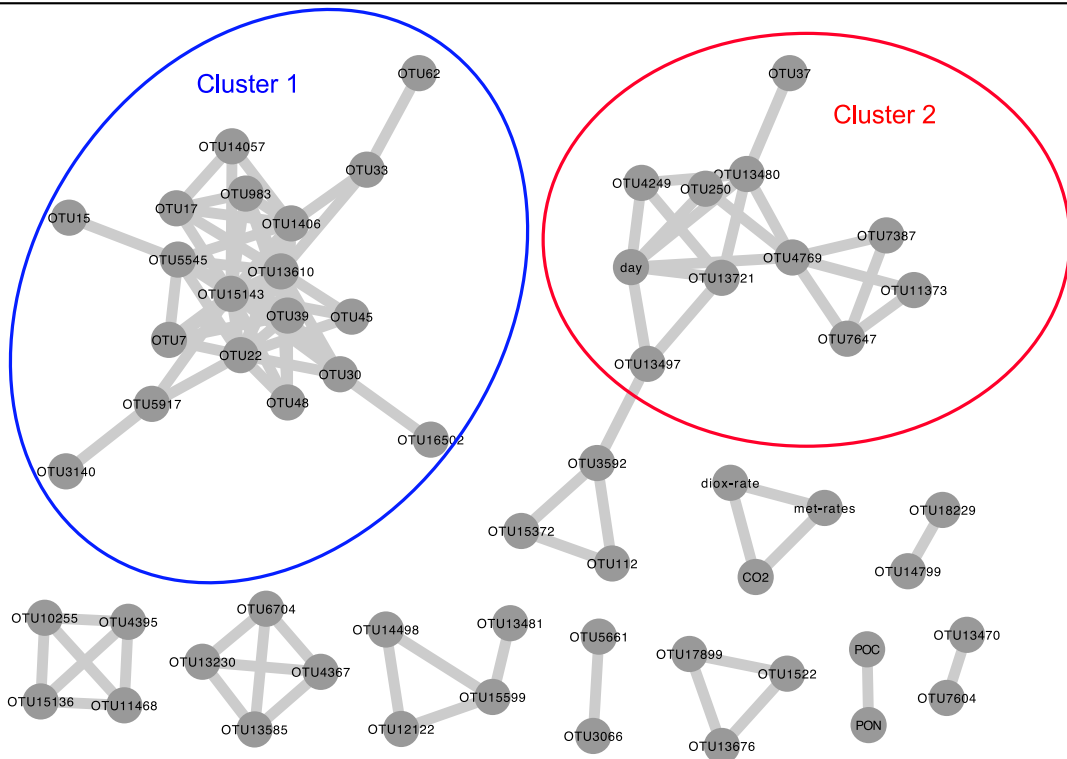

b) Cluster 1

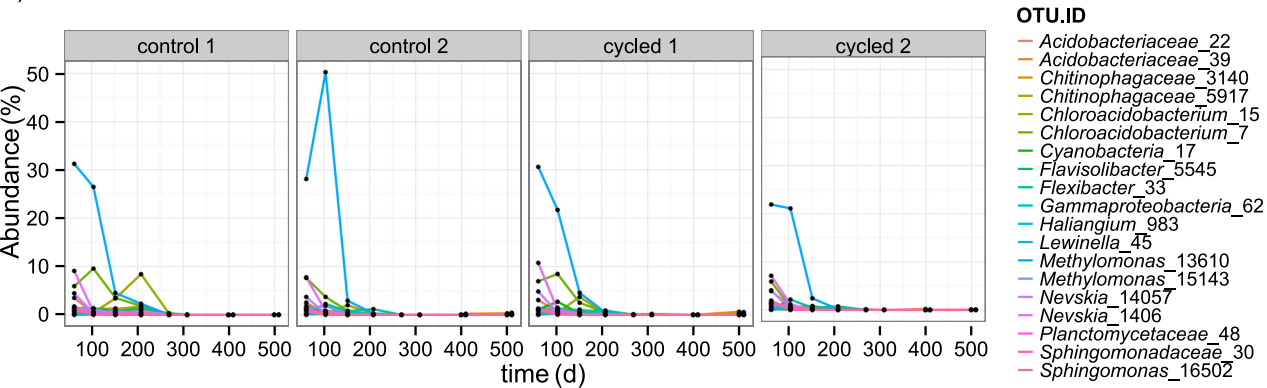

c) Cluster 2

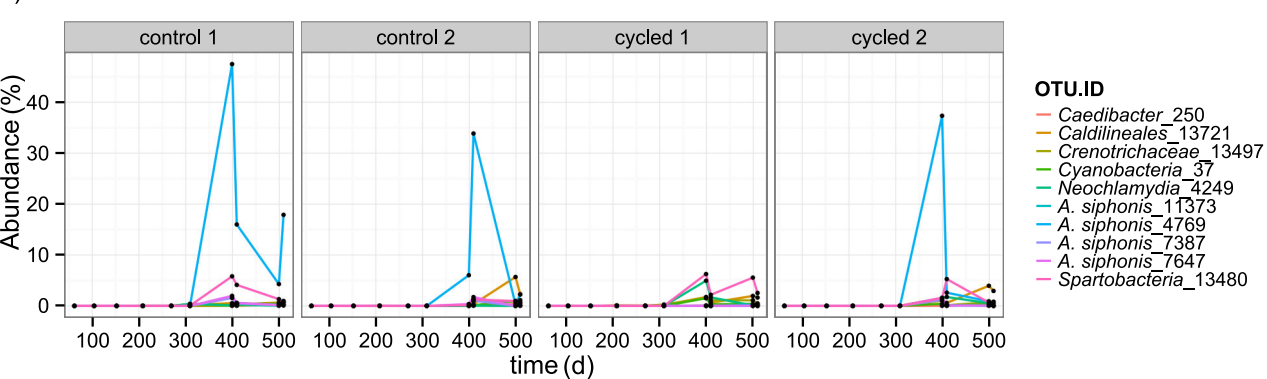

Supplement: Figure S7 [file sys005162053sf10.pdf]
