# Supplementary material for: Microbial Communities Are Well Adapted to Disturbances in Energy Input
Source: mSystems. 2016 Sep 13;1(5):e00117-16. doi: 10.1128/mSystems.00117-16 (PMC5080406; doi:10.1128/mSystems.00117-16)
Supplement: Text S1 [file sys005162053s1.docx]

**SUPPLEMENTARY INFORMATION**

**Supplementary materials and methods**

**Experiment set up.** Four 18L chemostats were inoculated in 2005 with unfiltered water samples collected from a cedar swamp (1L each) at 41.524224ºN, 70.655570ºW (MBL Swamp, 0 Oyster Pond Rd., Falmouth, Massachusetts, US) and supplemented with mineral media (see main text for composition). Chemostats were maintained in batch mode for approximately 5 years with continuous supply of methane plus air mixture (4.9% CH_4_, 19.6% O_2_, 0.03% CO_2_, balanced with N_2_) until the start of the experiment on 20 Aug 2010 (day 0). Methane was measured in-line with a non-dispersive infrared detector (601 NDIR, California Analytical Instruments, Orange, CA) and CO_2_ and O_2_ where measured in-line with a laser diode absorption spectrometer (O2Cap, Oxigraf, Sunnyvale, CA, USA). Gas from headspace of chemostats was sampled in a closed loop under the control of a multiport sample valve (STF selector, VICI Valco Instruments, Houston, TX, USA) and the gas was stripped of water with a Nafion drier (MD-Series, Perma Pure, Lakewood, NJ, USA) prior to gas analyzers. Gas analyzer drift was compensated by monitoring input gas composition. The methane gas mixture was also used during the experiment, sparged through ceramic diffusers (0652 Round Bottom Cup, Soilmoisture Equipment Corp., Goleta, CA, USA) at a gas flow rate of 20 mL min^-1^ (0 °C, 101.3 kPa) controlled by mass flow controllers (1179A and M100B MKS Instruments, Andover, MA, USA). During the CH_4_-off periods for the cycled chemostats in experimental phases III and IV, the methane plus air mixture was switched to solely air (20.95% O_2_, 0.033% CO_2_, balance N_2_). To ensure similar microbial communities, chemostats were cross-mixed at the beginning of the experiment and on days 10, 32, and from 123 to132. More details on the experimental apparatus can be found on the NSF ATB project web site ([http://ecosystems.mbl.edu/MEP]).

**Chemistry measurements.** Samples periodically withdrawn for the analysis of nitrate (NO_3_^-^), nitrite (NO_2_^-^), ammonia (NH_4_^+^), particulate organic carbon (POC) and nitrogen (PON), dissolved organic carbon (DOC) and nitrogen (DON) were filtered (precombusted glass fiber GF/F filter, Whatman), collected in acid washed Nalgene bottles or pre-combusted glass vials and frozen or refrigerated until further analysis. NO_3_^-^ plus NO_2_^-^ concentrations were determined colorimetrically on a flow injection analyzer (LachatQuickChem 8000, Lachat Zellweger Instruments, Milwaukee, WI, USA) using the cadmium-copper reduction method (1). NH_4_^+^ concentrations were determined colorimetrically using the phenolhypochlorite method modified for small sample size (2) using a Cary Spectrophotometer (Varian, Palo Alto, CA, USA). DOC and TDN were determined by high-temperature oxidation and infrared detection (TOC-Vcph for carbon and TNM-1 unit for total nitrogen, Shimadzu Scientific Instruments, Columbia, MD, USA), while POC and PON were measured by gas chromatography (2400 CHN elemental analyzer, Perkin Elmer, Waltham, MA, USA) as previously described (3). pH (Phoenix, Topac Instruments, Cohasset, MA USA) was measured and recorded every hour with a computer D230 data logger (Consort, Turnhout, Belgium). Gas cycling and all data acquisition were under computer control.

**Determination of microbial cell abundances.** To determine microorganism abundances, 60 mL were periodically withdrew and fixed in 4% formaldehyde (4 h at 4 °C), washed in phosphate buffered saline (PBS) and preserved in PBS-Ethanol mixture (1:1 (v/v)) at -20 °C. Eukaryotic microorganism concentrations were manually determined using fluorescent microscopy (4). For that, samples were filtered through a 0.22 μm membrane (Millipore, Billerica, MA, USA) and stained with DAPI. At least 100 fields of view per sample were counted on an Epifluorescent Microscope (Zeiss, N. Chesterfield, VA, USA). To determine prokaryotic abundances, fixed samples were sonicated for 10 minutes using a focused-ultrasonicator (20% duty factor, 300W peak incident, 50 cycles per burst, S220, Covaris, Woburn, MA, USA) to disrupt cell clumps. Samples were then stained with SYBRGreen I (Molecular Probes, OR, USA) for 10 min in the dark and analyzed on a flow cytometer (FACSCalibur Becton Dickinston, San Jose, CA, USA) equipped with a 488 nm, 15 mW Argon laser. Fluorescent beads (TruCOUNT medium control beads, Becton Dickinston, San Jose, CA, USA) were added to each sample to normalize the forward and side light scatters (FSC and SSC), and green (530nm) fluorescence signals, respectively. Prokaryotic cells were quantified using the plot of the green fluorescence vs. SSC while green fluorescence was used as the threshold parameter (WinMDI 2.9 software, The Scripps Research Institute [http://facs.scripps.edu/software.html]).

**Statistical analysis.** Library coverage was estimated using Good’s coverage (5). Even sequencing depth per sample was established by multiple rarefactions to the smallest sequencing depth. Bacterial diversity of microbial communities was estimated by Simpson diversity (1-D) and evenness (E) indices (6). Finally, Linear discriminant Analysis (LDA) effect size (LEfSe) (7) with a threshold of 2 on LDA score and 0.01 for the alpha values in the Krustal-Wallis test among classes, was used to determine the abundant OTUs with differential abundances between cycled and control treatments during experimental Phases III and IV.

A network was used to study dominant OTUs co-occurrence patterns and correlations with environmental parameters. Rare OTUs (relative abundance < 1%) were removed from the analysis although the total sum of rare OTUs abundances was kept for the network calculations. Next, all possible Spearman’s rank correlations (ρ) between dominant OTUs and between those OTUs and environmental parameters were calculated and the statistical significance was estimated with a combination of permutation and bootstrap (1000 iterations), using the Benjamini-Hochberg method to adjust for the false discovery rate. Robust and statistically significant co-occurrence events were defined as those with ρ > 0.60 and p-value < 0.05. Non overlapping algorithm HC-PIN was used to identify clusters of co-ocurring dominant OTUs (8). Network inference was performed using CoNet (9) and Cytoscape (10).

**REFERENCES**

1. **Wood E**, **Armstrong A**, **Richards F**. 1967. Determination of nitrate in sea water by cadmium-copper reduction to nitrite. J Mar Biol Assoc UK **47**:23–31.

2. **Solórzano L**. 1969. Determination of ammonia in natural waters by the phenolhypochlorite method. Limnol Oceanogr **14**:799–801.

3. **Knap A**, **Michaels A**, **Close A**, **Ducklow H**, **Dickson A**, **(eds)**. 1996. Protocols for the Joint Global Ocean Flux Study (JGOFS) core measurements. JGOFS Report Nr. 19, vi+170ppReprint of. UNESCO 1994, Paris.

4. **Porter KG**. 1980. The use of DAPI for identifying and counting aquatic microflora. Limnol Oceanogr **25**:943–948.

5. **Esty W**. 1986. The efficiency of Good’s nonparametric coverage estimator. Ann Stat **14**:1257–1260.

6. **Simpson EH**. 1949. Measurement of diversity. Nature **163**:688.

7. **Segata N**, **Izard J**, **Waldron L**, **Gevers D**, **Miropolsky L**, **Garrett WS**, **Huttenhower C**. 2011. Metagenomic biomarker discovery and explanation. Genome Biol **12**:R60.

8. **Faust K**, **Raes J**. 2012. Microbial interactions: from networks to models. Nat Rev Microbiol **10**:538–50.

9. **Faust K**, **Sathirapongsasuti JF**, **Izard J**, **Segata N**, **Gevers D**, **Raes J**, **Huttenhower C**. 2012. Microbial co-occurrence relationships in the human microbiome. PLoS Comput Biol **8**:e1002606.

10. **Shannon P**, **Markiel A**, **Ozier O**, **Baliga NS**, **Wang JT**, **Ramage D**, **Amin N**, **Schwikowski B**, **Ideker T**. 2003. Cytoscape : A Software Environment for Integrated Models of Biomolecular Interaction Networks. Genome Res **13**:2498–2504.
